# Six years of INSTAND e. V. sIgE proficiency testing: An evaluation of in vitro allergy diagnostics

**DOI:** 10.1007/s40629-016-0005-8

**Published:** 2017-01-18

**Authors:** N. Wojtalewicz, S. Goseberg, K. Kabrodt, I. Schellenberg

**Affiliations:** 1 Center of Life Sciences, AG IBAS, Hochschule Anhalt, Stenzfelder Allee 28, 06406 Bernburg (Saale), Germany; 2 INSTAND e.V., Düsseldorf, Germany

**Keywords:** INSTAND e. V., Round robin test, Allergy, Diagnostic test, In vitro diagnostic

## Abstract

**Background:**

Even though allergies are an important health issue, wide manufacturer-dependent differences in the detected amounts of allergen-specific IgE (sIgE) have repeatedly been found. These discrepancies hinder diagnostics and research into clinically significant cutoff points for life-threatening symptoms.

**Methods:**

To evaluate whether the reported differences have led to changes in diagnostic testing, we analyzed data from six years of round robin testing (RRT, also known as proficiency testing) at the Gesellschaft zur Förderung der Qualitätssicherung in medizinischen Laboratorien e.V.  (Society for Promoting Quality Assurance in medical Laboratories) for the important allergen sources bee venom, wasp venom, and birch pollen. The results of the four main suppliers of in vitro diagnostic sIgE testing were compared in a pseudo-anonymized form using overlay images of box plot graphs for the semiquantitative data and allergen class results. Coefficients of variation (CV) were obtained to study the development of interlaboratory comparability.

**Results:**

We found that the large differences between the manufacturer collectives remained constant between January 2010 and April 2015 without any real improvement. The CVs were good for two of the four analyzed suppliers, one was marginal and one above the quality level of 20%.

**Conclusion:**

The numerous publications that have found discrepancies in the sIgE results of the different suppliers did not change the status quo within the last six years. Unfortunately, this is unlikely to change until there is a characterized standard material with known values of sIgE.

## Introduction

Allergies have become more prevalent among global populations within the last 50 years [1]. In Germany, about 49% of adults have specific IgE antibodies (sIgE) against at least one of the 50 allergens tested [2].

Allergic rhinitis (14.8%) and asthma bronchiale (8.6%) have the highest lifetime prevalence in Germany [3]. These two diseases are often caused by outdoor allergens [4] such as birch pollen, against which 17.4% of allergy patients exhibit sIgE [2]. Allergies to hymenoptera venoms – for example from the honeybee or the yellow jacket (hereinafter referred to as bee and wasp venom) – represent a special allergic disease. This disease is far less prevalent, representing around 2.8% of cases [3]. However, despite this low rate of incidence, hymenoptera stings are reported to be the trigger in about 50% of all anaphylaxis cases in German-speaking countries [5]. This emphasizes the importance of having an accurate diagnosis for these allergens so that precautions can be taken before the first life-threatening reaction occurs.

The Gesellschaft zur Förderung der Qualitätssicherung in medizinischen Laboratorien (Society for Promoting Quality Assurance in medical Laboratories) (INSTAND e. V.) has managed round robin tests (RRTs, also known as proficiency testing) for in vitro allergy diagnostics since 1995 and is one of two officially appointed reference institutes in Germany. In 2003, it reported large discrepancies between the sIgE levels detected by systems from different manufacturers [6]. Since then, multiple studies addressing this problem have been published, comparing up to three different systems with a variety of serum samples. While some publications point out the differences between the testing methods [7–9], other laboratories report a good comparability for several allergen sources [10, 11]. A recent publication by Koch & Aberer evaluated the development of the allergen class results from the last 25 years and found good agreements between the laboratories [12]. But the good comparability of the allergen classes can be quite confusing, since they can sometimes mask high differences within the kU/L results [13]. The importance of the quality management in in vitro diagnostics is a frequently discussed topic, as shown by a recent review article by Kleine-Tebbe et al. [14]. This way, a constant attention is directed towards the sIgE-diagnostic and this should forward the effort for a better comparability.

To analyze possible changes within in vitro allergy diagnostics, we evaluated the data from six years of RRTs at INSTAND e. V. for the allergen sources birch pollen, bee venom, and wasp venom. This paper is the first presenting the chronological development of differences in detection levels. The analysis provides insights into semiquantitative results in kU/L, corresponding allergen classes, and interlaboratory comparability.

## Materials and methods

### Round robin testing procedures at INSTAND e. V.

Every participant receives five lyophilized serum samples for each RRT. Four samples are used to determine sIgE against inhaled allergens, food allergens, and hymenoptera venoms allergens, and one to determine total IgE. Three sera show high levels of sIgE, while two are dilutions from both sera for the determination of sIgE.

Each participant has to determine the allergen class and the sIgE concentrations (kU/L) of the defined allergen sources like birch pollen.

In order to evaluate sIgE concentrations, the results are sorted according to the manufacturer of the system. These groups of participants are referred to as “collectives”. The median for each collective is calculated after determining the clinically relevant range. Every participant whose results are within 25% of the median passes the evaluation and receives a certificate.

In terms of the allergen classes, the results are generally comparable among the manufacturers so that they can be evaluated for all participants within the RRT. Only when the results of one manufacturer collective lie within a completely different range, a separate median is formed for this collective. As with the concentration, every participant that is within 25% of the median passes the test and receives a certificate.

Furthermore, the coefficient of variation of the results within the evaluation is obtained for every collective in order to evaluate the interlaboratory variance for the serum sample.

### Data evaluation

For this publication, data from 17 RRTs carried out between 2010 and 2015 were analyzed for the allergen sources bee venom, wasp venom, and birch pollen. Between 140 and 460 participants took part in the different RRTs for the individual allergens. Each dataset was sorted according to manufacturer collective and then plotted as a box plot. This paper used all of the results except for the outliers that exceeded the highest calibration point of each collective by more than 20% (kU/L) or 0.5 allergen classes, respectively. The manufacturer code can be found in the RRT̕s supplementary booklet. Basic statistical analyses were performed using SigmaPlot 13 from Systat Software GmbH (Erkrath, Germany).

### Generation of images

The overlay images were generated using the Gnu image manipulator software 2.8.1.

## Results

This publication includes data on the three allergen sources birch pollen, bee venom, and wasp venom from 17 RRTs performed between January 2010 and April 2015. Both venoms were tested in all RRTs and birch pollen was included in 12 RRTs. During this time period, the number of participants increased from around 140 in 2010 to about 500 participants in 2015.

While the pass rate for the allergen class results remained relatively constant at around 90%, the pass rate for the semiquantitative sIgE determination rose from between 50 and 80% in 2010 to nearly 95% starting in early 2014. Nevertheless, this high pass rate for the semiquantitative analysis was only possible because the evaluation used manufacturer collectives due to the low comparability of the different detection systems (data not shown).

**Fig. 1:**
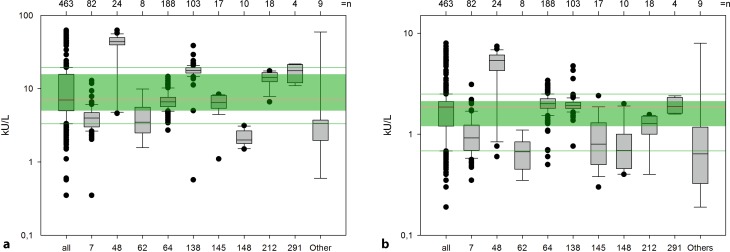
Manufacturer distribution for sIgE results of the allergen source birch pollen in the round robin test conducted in April 2015 for concentrated (**a**) and diluted (**b**) sample. The green box indicates 25–75% of all values combined. The red line marks the median of all values combined, and the green lines the general 10 and 90 percentiles. Manufacturers with fewer than four participants are summarized as “other”. The box indicates the 25–50% values, while the whiskers display the 10 and 90 percentiles of each collective

There was a great divergence in the detected amounts of sIgE, especially for birch pollen, as shown in the concentrated (Fig. 1a) and diluted sample (Fig. 1b) of the RRT from April 2015.

As expected, the combined results from all participants showed a very wide range of values (from 0 to ~65 kU/L). When the results are separated into supplier collectives, severe manufacturer-dependent variations emerge for the concentrated sample. While the results of the manufacturers F7, F62, F64, and F148 are between 0 and 15 kU/L, participants using a system from the manufacturer F138 detect amounts of around 0–40 kU/L and the systems from manufacturer F48 display a far wider variation of 5–65 kU/L for the same sample. A similar situation occurs for the diluted sample: While F7, F62, F145, and F148 have mostly low results, ranging from 0 to 2 kU/L, a majority of the participants using a system from F48 obtain results that are two to seven times higher (between 0.75 and 7.5 kU/L). Here, the results of the two main suppliers F64 and F138 reveal only small differences in contrast to the “low value group” (F7, F62, F145, F148).

Since F7, F48, F64, and F138 provide detection systems used by around 80% of all participants, this paper will focus on these four manufacturers with regard to the timelines and the analysis of the coefficient of variation (CV).

### Analysis of manufacturer distribution for birch pollen

**Fig. 2:**
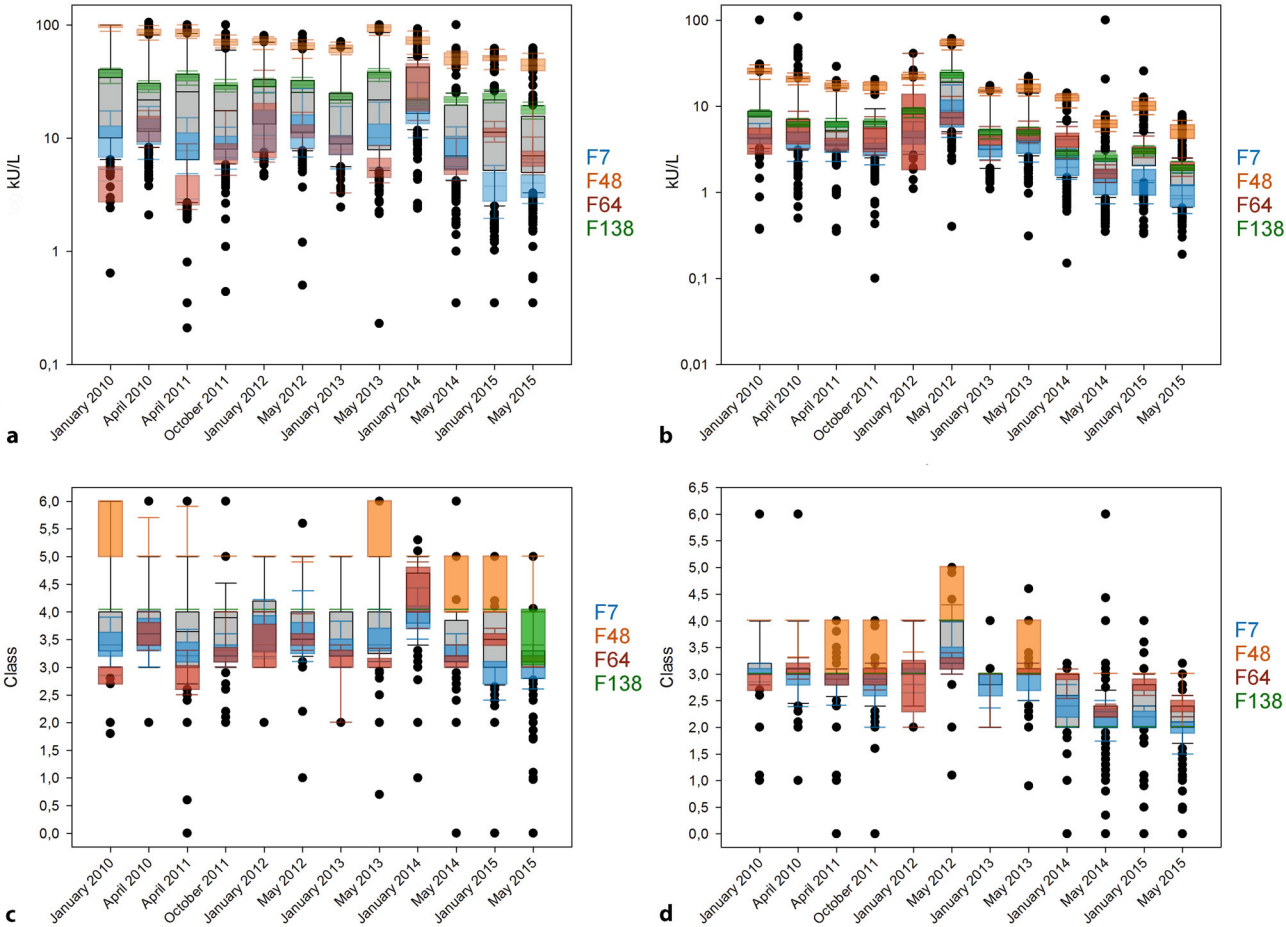
Timeline of the results of sIgE against birch pollen allergens from 2010 to 2015 for semiquantitative analysis of concentrated (**a**) and diluted sample (**b**) as well as for the RAST (radio-allergosorbent test) classes of concentrated (**c**) and diluted sample (**d**). The grey boxes display all results from the respective round robin test. The results obtained with the specific manufacturer collectives are illustrated as colored 10 to 90 percentile box plots. F7 is colored blue, F48 orange, F64 red, and F138 green

For birch pollen, the high values obtained using the detection systems from F48 did not only appear in April 2015, but can be observed for all RRTs for both the high level (Fig. 2a) and the diluted sample (Fig. 2b). With the concentrated sample there is consistently a large gap between the results of F48 and the next supplier collective F138. At the same time, the values obtained from manufactures F64 and F7 hardly ever exceed 20 kU/L and have a low distribution within the collective (except for F64 in January 2014). For the diluted sample, only F48 exhibits a noticeable difference to the other three suppliers in most of the times.

The large differences between the semiquantitative values also impact the classes, especially in the case of the concentrated sample (Fig. 2c). The differences for the diluted sample are not as severe and the results of all manufacturers collectives lie within 1.5 classes (Fig. 2d).

### Analysis of manufacturer distribution for bee venom

**Fig. 3:**
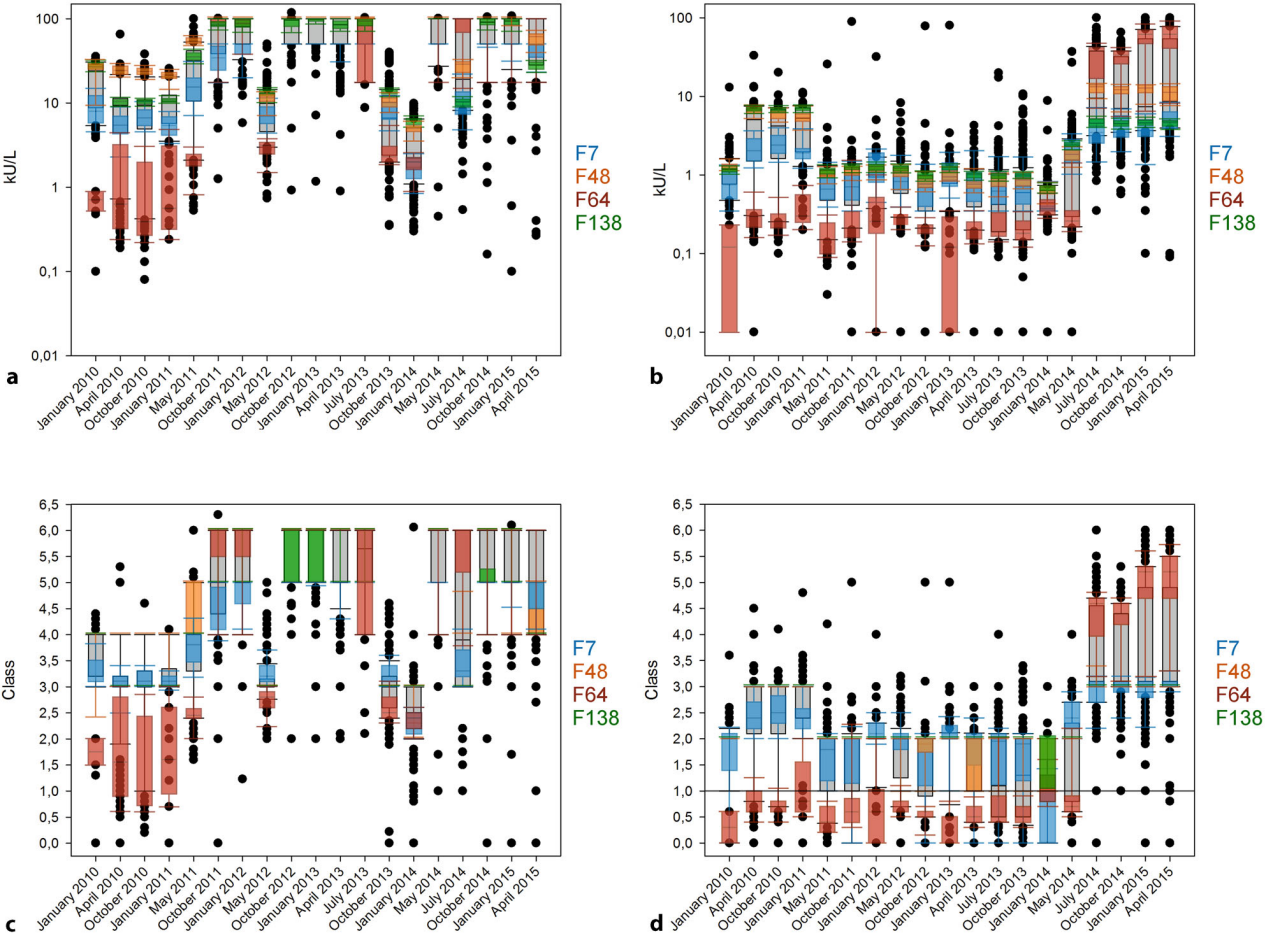
Timeline of the results of sIgE against bee venom allergens from 2010 to 2015 for semiquantitative analysis of concentrated (**a**) and diluted sample (**b**) as well as for the RAST (radio-allergosorbent test) classes of concentrated (**c**) and diluted sample (**d**). The grey boxes display all results from the respective round robin test. The results obtained with the specific manufacturer collectives are illustrated as colored 10 to 90 percentile box plots. F7 is colored blue, F48 orange, F64 red, and F138 green

Participants that used the systems provided by F48 often reached their systems saturation point (100 kU/L) and thus exhibit a low value distribution. In the RRTs from April 2010 to May 2011 they have the highest sIgE values of all distributors. They also detect high values in some other RRTs, but this time below those of the F138 collective. Interestingly, the F64 group displays high values when the general amount of sIgE is high, but has the lowest values when the general sIgE values (of all manufacturers combined) are below 40 kU/L. F138 tends to have higher values with the exception of the RRTs in July 2014 and April 2015 where they have even lower values than F7 (Fig. 3a). Within the classes, F7 and F64 mainly display a value distribution, while F48 and F138 often detect one class (Fig. 3c).

sIgE results for the RRTs in the diluted sample are relatively low between January 2010 and May 2014. Later, the values as well as the range increase significantly. F64 is the only collective that displays high values from July 2014 onwards (Fig. 3b).

Once the quantitative results are converted into the allergen class system, the low F64 sIgE values are mostly below the cut-off point for an allergy diagnosis before they suddenly rise in July 2014 and range at least one class above the other three manufacturers (Fig. 3d). As for the concentrated sample, F48 and F138 mostly detect one class, while F7 and F64 exhibit a value distribution (Fig. 3d).

### Analysis of manufacturer distribution for wasp venom

**Fig. 4:**
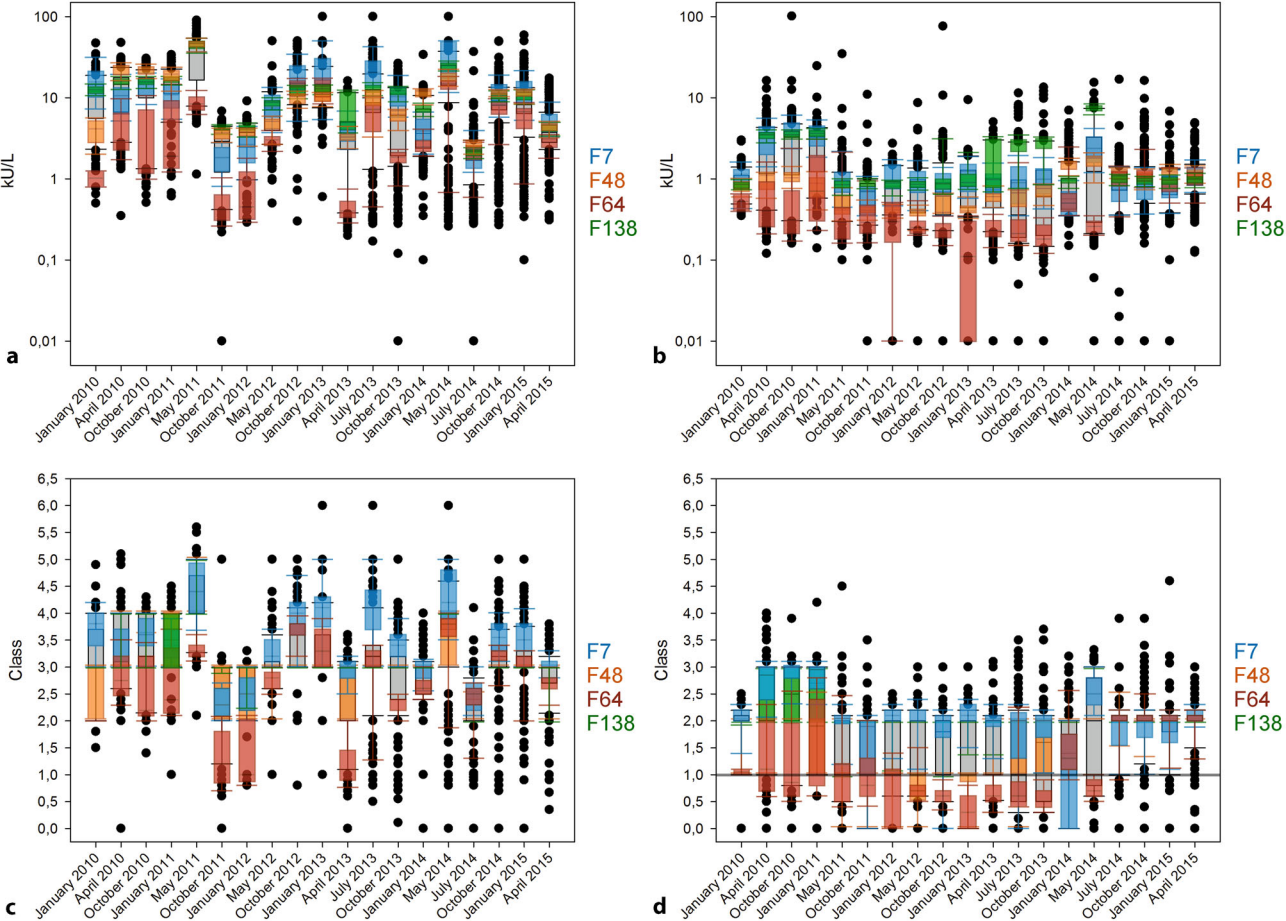
Timeline of the results of sIgE against wasp venom allergens from 2010 to 2015 for semiquantitative analysis of concentrated (**a**) and diluted sample (**b**) as well as for the RAST classes of concentrated (**c**) and diluted sample (**d**). The grey boxes display all results from the respective round robin test. The results obtained with the specific manufacturer collectives are illustrated as colored 10–90 percentile box plots. F7 is colored blue, F48 orange, F64 red, and F138 green

In contrast to the other two allergen sources, sIgE levels for wasp venom hardly exceed 30 kU/L. Only two of the 19 RRTs detect sIgE up to 50 kU/L (Fig. 4a).

Another difference is the value distribution of the F7 collective; it exhibits the highest sIgE concentrations for both samples in most of the RRTs except for those between April 2010 and January 2012. Surprisingly, F48 only displays its highest values in the concentrated sample from April 2010 to May 2011 and in the diluted sample in October 2014. In the other RRTs, the collective’s values fall between those of F64 and F7/F138. F64 shows the lowest results for the concentrated sample in all RRTs. In the diluted sample, there are also low values for the RRTs until July 2014, but later they are comparable to F7 (Fig. 4a, b).

If the semiquantitative results are converted into allergen classes, F138 once again mainly shows results within one class. F48 displays a greater distribution and F64 exhibits the lowest results once again. F64 is the only supplier with a high incidence of results below allergen class 1 in the diluted sample, thus indicating no allergy. After May 2014, there is no general manufacturer-dependent tendency to have allergen classes below class 1 (Fig. 4c, d).

### Quality development for in vitro allergen diagnostics for bee venom

The wide range in box plots within the diagrams above, especially for the semiquantitative analysis, indicates low interlaboratory comparability for some supplier collectives. The CVs were exemplarily generated for the semiquantitative sIgE results for bee venom (Table 1) and are comparable to the other two allergen sources. CVs that exceed the critical quality mark of 20% [6] are highlighted in red.

**Table 1:** Chronological development of the coefficients of variation (CVs) for semiquantitative analysis of bee venom for the concentrated (High) as well as the diluted sample (Low). Values that exceed 20% are highlighted in bold

Round robin test	CV [%] F7		CV [%] F48		CV [%] F64		CV [%] F64-4		CV [%] F64-6		CV [%] F138	
–	High	Low	High	Low	High	Low	High	Low	High	Low	High	Low
January 2010	**46**	**177**	**33**	14	**37**	**142**	0	–	**37**	**142**	12	**25**
April 2010	**42**	**54**	14	14	**102**	**48**	**36**	**30**	**54**	**58**	16	15
October 2010	**37**	**41**	15	15	**120**	**120**	**37**	**134**	**56**	**57**	11	9
January 2011	**38**	**75**	**25**	**28**	**131**	**54**	**80**	**45**	**40**	**22**	8	9
May 2011	**57**	**277**	10	15	**35**	**57**	**57**	15	**28**	**61**	**23**	13
October 2011	**36**	**57**	19	**24**	**43**	**115**	12	**39**	0	**34**	17	**420**
January 2012	**25**	**52**	0	13	**28**	**97**	–	–	0	**78**	14	**43**
May 2012	**83**	**86**	19	**22**	**27**	**263**	**31**	7	**21**	**263**	**22**	11
October 2012	7	**61**	0	**21**	**23**	**35**	0	12	0	**36**	18	**445**
January 2013	5	**56**	0	18	0	**90**	–	–	0	**90**	16	**400**
April 2013	**25**	**85**	0	**28**	**23**	**60**	15	**74**	0	**44**	14	11
July 2013	0	**68**	16	**24**	**47**	**343**	11	**226**	1	**451**	13	13
October 2013	**39**	**148**	**28**	20	**57**	**92**	**26**	**44**	20	**108**	14	92
January 2014	**44**	**191**	16	18	**46**	**46**	**86**	**43**	32	**44**	12	10
May 2014	15	**42**	12	175	**41**	**138**	3	**68**	12	**64**	0	12
July 2014	**60**	**55**	17	15	**44**	**53**	**24**	**34**	10	**32**	16	14
October 2014	**21**	**107**	18	**23**	**40**	**45**	2	**30**	11	19	15	14
January 2015	**26**	**46**	14	17	**40**	**52**	8	19	2	**22**	13	**174**
April 2015	**29**	**37**	28	**23**	**37**	**51**	5	**30**	7	**31**	20	**22**

F7 often achieves CVs that are higher than 20% for most of the concentrated samples and even above 30% for the diluted samples. Only four RRTs are able to achieve CVs below the critical mark.

Collective F64 exhibits similar and even higher CVs, but only as long as the results obtained by the two different systems F64-4 and F64-6 are combined. Once they are divided, the CVs strongly decrease after May 2011 for the concentrated sample, but hardly ever do this for the diluted one.

The only suppliers that mostly display CVs below 20% for both samples are F48 and F138 with a few spikes in some years.

All manufacturer collectives show spikes above 100% for several RRTs and even some CVs of 0%. The low values correlate to the RRTs where high amounts of sIgE for bee venom are detected (cp. Fig. 3a, b) and the supplier collectives reach the saturation point of the systems used.

When the IgE values are displayed in allergen classes, all of the manufacturers analyzed have CVs below 20% (data not shown).

## Discussion

After the initial reports about the wide system-dependent differences in the amounts of sIgE detected, improvements in the allergy diagnostics became necessary. Many attempts have been made since then, from both a regulatory and a supplier perspective, including mandatory participation in RRTs twice a year [15] and the generation of calibration samples in line with the WHO standard 75/502.

The aim of this study was to align the different detection systems in order to improve in vitro allergy diagnostics and general research. Therefore, the data from the last six years of INSTAND e. V. were used to examine the status quo. Unfortunately, there remains a stark difference between the results of the four most prominent suppliers for the important allergen sources birch pollen, bee venom, and wasp venom.

For the outdoor allergen source birch pollen, the collective F48 detects significantly higher values than the other three, while F7 and F64 always display low results for sIgE. We are able to show that within 2010 and 2015 no efforts have been made to align results.

The reasons for these differences remain unchanged: The use of different analytical systems is one factor, but the major reason is most probably the vast complexity of allergen extracts and sIgE binding epitopes on the allergens. For example, the major birch pollen allergen Bet v1 indicates at least four to six different isoforms in the pollen of an individual tree [16]. Using recombinant proteins could present a more consistent basis for generating assays even though different isoforms of rBet v1 also exist (e. g., [17–19]). As long as the suppliers are not using the same mixture to generate and calibrate the system, the current detection differences are likely to remain.

The use of single proteins for the systems as only indicators for sensitization is also not recommended since there are regional variations in the reactivity to different birch pollen proteins within Europe. Movérare et al. was able to show that around 98% of patients sensitive to birch pollen in Sweden, Finland, and Austria had sIgE against Bet v1, while the percentage in Switzerland and northern Italy was only 70% [20]. Nevertheless, a mixture of different (recombinant) pure proteins might improve the general comparability, even though regional specialties should be considered for different mixtures. Furthermore, the general allergen amounts should be kept in mind, to prevent false positive results.

Hymenoptera venoms are a special allergen source, not only because of the high risk of life-threatening anaphylaxis, but also due to the elevated number of negative results within the diagnostic workup. About 15–20% of the patients with both a positive medical history and skin test have negative in vitro results. Furthermore, the sIgE values do not correlate to the severity of the clinical symptoms [21–23]. The skin tests results might be considered more stable, but there are negative results, even when symptoms occur. Golden et al. suggest that 30% of patients allergic to hymenoptera venom might have a negative skin test result because of the limited sensitivity of the reagents used [24].

A negative in vitro result in an allergy diagnosis is defined as an sIgE level below 0.35 kU/L (allergen class 1). Among the INSTAND e. V. data, only supplier F64 showed an overwhelming tendency to produce sIgE results below this critical mark for both bee and wasp venom between January 2010 and May 2014. The three other manufacturer collectives detected higher amounts and thus a sensitization. After May 2014, the detection levels from F64 suddenly rose and were the highest for bee venom and equivalent to F7 for wasp venom. This indicates that the manufacturer has made some changes to its systems. This alteration could be due to the use of new venom allergen standards. There are reports that major allergens are missing or underrepresented in the hymenoptera venom standards, e. g., Ves v5 in the wasp venom extract [25] and Api m10 in the bee venom standard [26]. The importance of these insufficiently represented allergens was shown by Vos et al., who were able to increase sensitivity to an assay from 84.4 to 96.8% by spiking the extract with recombinant (r) Ves v5 [25].

F48 also had one noticeable shift in the general detection tendency: From January 2010 until May 2011 they displayed the highest sIgE values for both hymenoptera venoms. In terms of the bee venom, there was a huge gap between the F48 results and the next in line. However, after October 2011, they detected average results.

The high values for both manufacturers F48 and F64 were possibly due to more potent IgE-binding extracts or because of an enhanced cross-reactivity of their standards. There are high similarities between different proteins within the hymenoptera venoms, for example, between the honeybee protein Api m2 and the wasp protein Ves v2 [27]. Furthermore, cross-reactive carbohydrate structures (CCDs) present a serious problem in the hymenoptera venom allergy diagnostic [28, 29] and they can react to different hymenoptera venoms as well as to other allergen sources [30–32].

The CCD dependent cross-reactivity can be eliminated by the use of recombinant proteins produced in bacteria [33]. However, CCD-independent cross-reactivities still remain, as shown by different research groups (e. g., [34–36]).

Koch & Aberer evaluated the allergen class results for sIgE and they reported a very good comparability between the participants over the last 25 years in Austria (and since 2006 in neighboring countries). These results are mainly due to the fact that only the allergen classes were evaluated. Furthermore, their criteria for a “false” result was less strict than those of INSTAND e. V.: It was only considered “false” if it differed more than two classes from the overall results or was negative, while the mainstream showed a sensitization. Especially within the higher classes (above 3), the value span is quite high; so many differences, as we could observe within our analysis, simply vanished due to the focus on the allergen classes [12]. The importance of the evaluation of the semiquantitative values was pinpointed by Wood et al. as well: They compared the results from two established laboratories within the USA and showed high differences between the three methods evaluated, once it comes down to the semiquantitative results [5].

This further highlights the semiquantitative basis of the detection systems as a general problem in in vitro allergy diagnostics. This means it is currently impossible to determine which collective detects the “almost true sIgE values”. The development of a fully characterized serum sample or a “serum-like” sample with defined spikes of sIgE is one possible solution to this problem. Chimeric antibodies might be a big help to provide these spikes, as shown by Wood et al., who used this kind of antibodies to evaluate the origin of the differences between the results from various manufacturers. Their findings also indicated a low comparability of the detected values for sIgE to the WHO standard, since the sIgE-results provided by some systems show a huge deviation from the total IgE results [5]. So the question arises whether the calibration curves are correct? A further advantage of an “artificial serum sample” with chimeric antibodies might be that there is no interference to epitope specific IgG antibodies.

Once the true values are known, suppliers who detect the wrong result have no other choice but to correct their system. By using monoclonally engineered sIgE, one could also enlarge the allergen source pool to the other hymenoptera species where no allergen extracts presently exist. Currently, a sensitization to the bumblebee and hornet are most likely to be detected as an allergy to bee venom due to the high cross-reactivity [28].

While the analysis of the RRTs not only reveals that there is still a poor consensus between the different manufacturers, it also provides insight into the performance of the individual laboratories as well as the robustness of the assays. A comparison of the CV for the individual collectives is a good indicator of interlaboratory comparability.

Even though they showed higher sIgE values than most other suppliers, the CVs of F48 and F138 were around, or even below, the “critical” quality mark of 20%, with the exception of a few spikes, especially within the diluted sample. This tendency remained constant over the six year study period, indicating a generally robust system as well as a good management of production quality.

F64 showed high CVs that usually diminished once the results were divided into the two different detection systems. Within the concentrated sample, there were very low CVs, including zero values. These low CVs correlated with the RRTs that displayed a general tendency for high sIgE values (cp. Fig. 3a) and where the F64 collectives mostly reached their saturation points. Once the diluted sample was analyzed, only few RRTs achieved CVs below 20% for the two different F64 systems, indicating an unstableness within lower sIgE results.

F7 showed high CVs in general with a few exceptions where all participants reached the highest calibration point of their detection system. Within the diluted sample, there was no RRT with a CV below 30%. This might be a promising tendency, since the CVs dropped during 2015, but such low values had occurred from time to time in the past as well. A constant observation is needed to ensure that this good tendency remains constant in the future.

The high results for the CVs of F7 and F64 (>20%) were relatively surprising since other publications have reported CVs of 20% and below for different systems [6, 7]. The high values presented in this publication might be due to the fact that the data includes all results provided by the participants of the respective RRT. Only values that exceeded the calibration by more than 20% were excluded since they were more likely to be transfer errors. Nevertheless, there were still outliers present in some RRTs that were up to 30 times higher than the median. A high number of participants, as described in this publication, increases the probability of those outliers.

The results of the allergen classes presented here were expected to be good, since a wide range of high values were only exhibited in one class, so even a difference of 20 kU/L would not have changed the class.

The manufacturer-dependent differences are not only present for the birch pollen and the hymenoptera venoms, but also for food allergens [37] and other inhaled allergen sources like house dust mite and cat epithelium [38].

## Conclusion

In summary, this publication shows that there are still large supplier differences, most likely due to the use of complex allergen extracts instead of defined allergen compounds. The use of defined proteins, like recombinant constructs, might not solve this problem since different isoforms exist here as well. Thus, further research is needed. The quality of the performance is good in the case of F48 and F138, but there is a dire need of improvement in the case of F7 and F64. INSTAND e. V. will contact these suppliers to address the problem and offer help in finding a solution.

## Abbreviations

CCD: Cross-reactive carbohydrate structure
CV: Coefficient of variation
INSTAND: Gesellschaft zur Förderung der Qualitätssicherung in medizinischen Laboratorien (Society for Promoting Quality Assurance in medical Laboratories)
RfB: Reference Institute for Bioanalytics
RRT: Round robin test
sIgE: Specific IgE
